# Directly lighting up RNA G-quadruplexes from test tubes to living human cells

**DOI:** 10.1093/nar/gkv1040

**Published:** 2015-10-17

**Authors:** Shujuan Xu, Qian Li, Junfeng Xiang, Qianfan Yang, Hongxia Sun, Aijiao Guan, Lixia Wang, Yan Liu, Lijia Yu, Yunhua Shi, Hongbo Chen, Yalin Tang

**Affiliations:** 1 National Laboratory for Molecular Sciences, Center for Molecular Sciences, State Key Laboratory for Structural Chemistry of Unstable and Stable Species, Institute of Chemistry Chinese Academy of Sciences, Beijing, 100190, P. R. China; 2 University of the Chinese Academy of Sciences, Beijing, 100049, P. R. China

## Abstract

RNA G-quadruplexes (G4s) are one of the key components of the transcriptome that act as efficient post-transcriptional regulatory elements in living cells. To conduct further studies of the unique biological functions of RNA G4s, techniques need to be developed that can efficiently recognize RNA G4 structures under various conditions, in fixed cells and living cells, as well as *in vitro*. This paper presents the development of such a method, a new technique using a cyanine dye called CyT, which can detect both canonical and non-canonical RNA G4 structures from test tubes to living human cells. The ability of CyT to distinguish between G4 and nonG4 RNA offers a promising tool for future RNA G4-based biomarker discovery and potential diagnostic applications.

## INTRODUCTION

The nucleic acid structures G-quadruplexes (G4s), which are formed from G-rich sequences by stacking of Hoogsteen bonded G-quartets (1), are prevalent throughout the human genome and transcriptome (2,3). It has been confirmed that both DNA and RNA G4s are formed *in living cells* and are associated with many important cellular processes (4–8). As a number of G4s have been found associated with human diseases, such as cancer and diabetes, they have been viewed as emerging diagnostic and therapeutic targets (8–12). While most early studies have been focused on DNA G4s, there is a rapidly growing interest in investigating RNA G4s. Accumulating evidence shows that RNA G4s play important roles in many biological processes such as pre-mRNA splicing and polyadenylation, RNA turnover, mRNA targeting and translation (13). Currently, a great number of putative RNA G4 sequence motifs have been identified within the human transcriptome (14,15). As RNA G4 is one of the key motifs in the transcriptome (16), techniques that could efficiently recognize RNA G4 structures are crucial for studying the biological functions and impacts of RNA G4s.

Currently, there are several techniques used for detecting RNA G4 structures. X-ray crystallography and nuclear magnetic resonance (NMR) are the most accurate techniques and have identified high-resolution structures of RNA G4s (17,18). These techniques are most suitable for studying RNA G4 structures comprehensively. Circular dichroism (CD) is extensively used for monitoring G4 formation. The G4 structures exhibit characteristic CD spectral patterns (19). However, the sensitivity of the technique is limited, and the CD spectra may be difficult to interpret in the presence of different forms of nucleic acids. Recently, the presence of RNA G4 structures in human cells was visualized by using an engineered structure-specific antibody (4,5). However, antibodies cannot cross the cell membranes of living cells, which therefore limits their use for directly detecting RNA G4s in living cells. In-line probing is a useful technique to accurately identify guanine bases that comprise the G-tetrad in RNA G4s (20). Given that in-line probing requires a series of procedures, such as DNA template design, polymerase chain reaction (PCR) amplification of the template, *in vitro* transcription and radioactive 5′-end-labeling, the technique may be more suitable for studying nucleotide locations in RNA G4s, and not for *in situ* RNA G4 detection. Recent research has shown that nucleic acids that do not obey the classical description of sequences G_2+_N_L1_G_2+_N_L2_G_2+_N_L3_G_2+_ could also form G4 structures (21–24). However, there are only few reports of molecular probes that can recognize and light up these non-canonical RNA G4 structures. Although all of these above techniques are useful for the detection of RNA G4s, there is still an urgent need for efficient RNA G4 detection methods. Molecular probes which can specifically recognize RNA G4 structures may provide a simple and reliable way for RNA G4 detection.

Until now, considerable efforts have been focused on finding probes used for detecting DNA G4s (25–30), whereas only limited work has been carried out to search for RNA G4-recognizing probes. Some of the known DNA G4 probes, such as Thiazole Orange (TO), TmPyP4, ETC and pyridostatin (PDS), exhibit good structural selectivity for DNA G4s (31–34). Given the structural similarities between DNA and RNA G4s, it could be hypothesized that DNA G4 ligands may also show similar effects on RNA G4s; however, studies on these DNA G4 ligands showed that this is not the case. For instance, the porphyrin TmPyP4 can stabilize DNA G4 structures but destabilize RNA G4 structures *in living cells* (35,36); TO shows good selectivity on DNA G4s toward different DNA forms (37) but exhibits poor selectivity on RNA G4s toward other RNA forms (described later). Thus, these results show that currently available DNA G4 probes may not be suitable for detecting or recognizing RNA G4s. Moreover, another issue is that many of the RNA G4-binding molecules can also interact with other RNA forms, such as single-stranded, double-stranded and hairpin motifs, thus exhibiting poor selectivity (38). Small molecules emitting fluorescence that selectively binds RNA G4s at sites, which are not present in other RNA motifs, may be used as RNA G4 detectors.

Fluorescent cyanine dyes have been used extensively for decades as non-radioactive probes for detecting nucleic acids (39,40). Our previous work revealed a series of cyanine dyes that selectively recognize DNA G4 structures with different mechanisms (41–43). For instance, the ETC molecule recognizes intramolecular DNA G4 by an end-stacking mode, while the DMSB molecule recognizes the intermolecular DNA G4 by a dual-site simultaneous binding mode (41,43). Given that a number of cyanine dyes share many important spectroscopic and physical properties that facilitate nucleic acid detection, such as high molar absorptivity, low intrinsic fluorescence and large fluorescence enhancements upon binding to target nucleic acids, cyanine dyes provide us with a valuable candidate pool for discovering novel fluorescent probes for RNA G4 detection. Our group recently developed the G-quadruplex ligands database (G4LDB) (44), which aimed specifically at designing and screening G4 ligands. By using the RNA G4 screening models in G4LDB (entry ID 3MIJ), hundreds of cyanine dyes were evaluated and scored on their binding abilities with RNA G4s. Then, the cyanine dyes ranked in the top 10% were further evaluated by fluorescence assays. Thus, we discovered CyT, a fluorogenic cyanine dye with high RNA G4-sensing ability and specificity, which showed over 1000-fold fluorescence enhancement in the presence of RNA G4s, compared to less than 25-fold fluorescence enhancement observed in the presence of non-G4 RNAs.

**Scheme 1.**
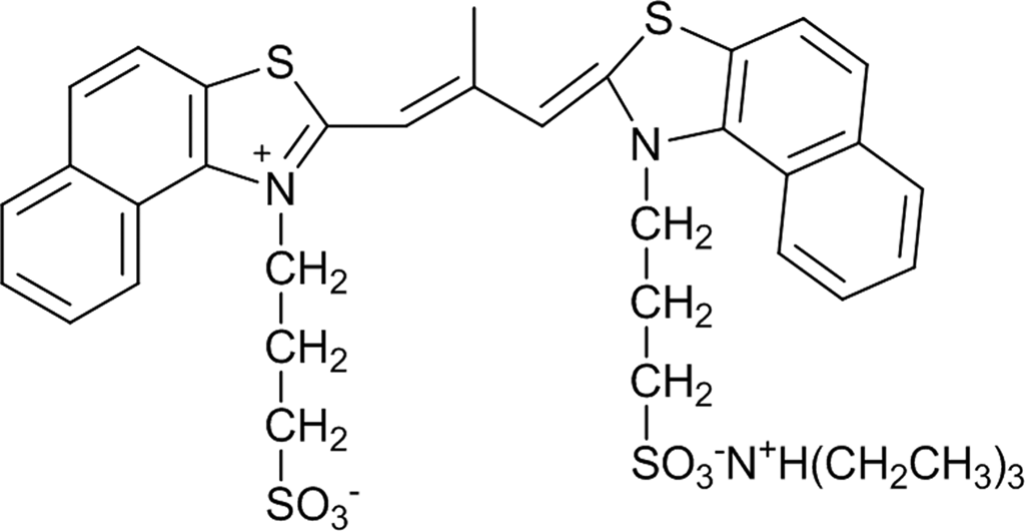
Molecular formula of the cyanine dye CyT.

This report describes the specific recognition ability of the fluorogenic cyanine dye CyT (Scheme 1) for RNA G4 structures among other RNA motifs through its fluorescence light-up in the longer wavelengths of the visible region. Here we describe the optical properties of CyT upon binding to different RNA motifs in different conditions. We also show that CyT can be used in detection for polyacrylamide gel electrophoresis (PAGE) and microscopy in fixed and living human cells.

## MATERIALS AND METHODS

### Oligonucleotides and compounds

Oligonucleotide sequences shown in Table 1 were synthesized by RiboBio Co. Ltd. Transfer RNA (Ribonucleotide acid; transfer from bovine liver; R4752) was purchased from Sigma-Aldrich. RNA stock solutions were prepared by dissolving oligonucleotides directly in 20 mM Tris-HCl pH 7.0, 40 mM KCl, and annealing in a thermocycler (first heated at 90°C for 2 min, then cooled down slowly to room temperature). The cyanine dye CyT was synthesized according to Hamer and Fichen's methods (45,46), and its purity was assessed by mass spectrometry, elemental analysis and NMR (Supplementary Information Part 1). The TO dye was obtained from Sigma-Aldrich. Stock solutions for CyT and TO were prepared by dissolving CyT and TO in methanol and water, respectively. RNase T1 was purchased from Invitrogen. All other chemicals were of analytical reagent grade and used without further purification. Ultrapure water, prepared by Milli-Q Gradient ultrapure water system (Millipore), was used in all experiments.

**Table 1.** Oligonucleotides used in this work

Name	Type/origin	Sequence (from 5′–3′)	Ref.
Tel22	Canonical G4-TERRA	AGGGUUAGGGUUAGGGUUAGGG	(49)
VEGF	Canonical G4–5′-UTR	GGAGGAGGGGGAGGAGGA	(50)
TRF2	Canonical G4–5′-UTR	CGGGAGGGCGGGGAGGGC	(51)
BCL-2	Canonical G4–5′-UTR	AGGGGGCCGUGGGGUGGGAGCUGGGG	(52)
NRAS	Canonical G4–5′-UTR	GGGAGGGGCGGGUCUGGG	(53)
Bulges-TB1	Non-canonical G4	UUGUGGUGGGUGGGUGGGU	(21)
Spinach	Non-canonical G4	GCAGCCGGCUUGUUGAGUAGAGUGUGAGCUCCGUAACUGGUCGCGUC	(22)
		GACGCGACCGAAUGAAAUGGUGAAGGACGGGUCCAGCCGGCUGC	
tRNA-Ala fragment	tRNA fragments	GGGGGUGUAGCUCAGUGGUAGAGCGCGUGC	(54)
tRNA-Cys fragment	tRNA fragments	GGGGGUAUAGCUCAGUGGUAGAGCAUUUGA	(54)
Tel22-mut	Mutation	AGUGUUAGUGUUAGUGUUAGUG	−
NRAS-mut	Mutation	GUGAGUUGCGUGUCUGUG	−
tRNA	Transfer RNA		−
ssAf17	Single strand	CUGAGUUGUAUAUAUUCG	−
ssAf22	Single strand	UGAGCUUAAUUGUAUAUAUUCG	−
HP18	Hairpin	CAGUACAGAUCUGUACUG	(4)
dsNC	Duplex	UUGUGCUACCAGGAGUCUG	−
		CAGACUCCUGGUAGCACAA	
3-WJ	Three-way junction (entry ID 619)	AGCGCAACCCC	(55)
		UCGUCAGCU	
		GGGACGACGU	
4-WJ	Four-way junction (entry ID 1173)	GCGC	(55)
		UUUAGC	
		GAGGUCCU	
		AGAA	

### Absorption spectroscopy

Absorption spectra were acquired with UV-1601PC at room temperature using a quartz cuvette with a path length of 10 mm. The molar extinction coefficient of CyT was determined by the commonly used method described by Sun and Goldys (47). Absorption titration experiments were performed by increasing RNA G4 concentrations from 0.125 to 4 μM, with the CyT concentration fixed at 2 μM.

### Fluorescence spectroscopy

Fluorescence spectra were acquired at 25 ± 1 °C, using a Hitachi F-4500 spectrophotometer equipped with a temperature-controlled circulator. A 10-mm path length quartz cuvette was used in all experiments. For fluorescence measurements, both excitation and emission slits were 5 nm, and the scan speed was set at 240 nm/min. CyT was titrated with RNA G4 for measurement of the binding constants, with the fluorescence intensity at 570 nm plotted as a function of the RNA concentration. The data were fitted according to a 1:1 binding model. The titration experiments were performed by increasing RNA G4 concentrations from 0.125 to 4 μM. CyT at the specified concentration was added to solutions of different RNA concentrations, with gentle stirring for 10 min, and then the samples were kept in darkness for 2 h before measurements were taken. Fluorescence quantum yield values were acquired by the method described previously (48).

### Circular dichroism spectroscopy

CD spectra were recorded on a JASCO J-815 spectrophotometer equipped with a temperature-controlled circulator. CD measurements were carried out at 25 °C in the wavelength range of 220–350 nm, using a response time of 1 s, a step size of 1 nm and a 2 nm bandwidth. The scanning speed of the instrument was set at 200 nm/min, with an average of three scans. A 10-mm path length quartz cuvette was used in all experiments.

### Polyacrylamide gel electrophoresis

PAGE was performed in 1× TBE buffer solution (0.09 M Tris-boric acid and 0.002 M EDTA) with 12% native gels. Oligonucleotides (5 μM) were loaded on the gel, and electrophoresis was run at 64 V for 2 h at room temperature. After electrophoresis, the gel was stained using either 20 μM CyT in Tris-K^+^ or 1× SYBR Gold, under constant agitation for 15 min, then lightly rinsed with water and visualized using GE Typhoon 9400 Trio. Fluorescence images with excitation wavelength of 532 nm were recorded using the emission filters of 575 nm (for SYBR Gold) and 610 nm (for CyT).

### Cell cultures and microscopy

A549 cells were cultured in Dulbecco's Modified Eagle's Medium (DMEM) contain 5% fetal bovine serum (FBS), streptomycin (0.1 mg/mL) and penicillin (100 U/mL), under 5% carbon dioxide at 37 °C. Cells grown on Petri dish were fixed for 5 min in cold methanol and 4% formaldehyde, rinsed twice with phosphate-buffered saline (PBS), and then permeabilized with 0.1% Triton X-100/PBS for 30 min at 37 °C. For enzymatic treatments, cells were incubated denaturing buffer and RNase T1 for 5 h at 37 °C after permeabilization. After treatment, cells were incubated with the CyT probe (5 μM) for 5 min. Cellular nuclei were stained with 4′,6-diamidino-2-phenylindole (DAPI), before visualization under a confocal laser scanning microscope (CLSM) (OLYMPUS FV1000-IX81) equipped with an oil immersion 100X objective. CLSM images of DAPI and CyT were collected under excitation at 408 nm and 559 nm, respectively. For living cells, the CyT probe was incubated with the cells for 24 h and visualized, without washing, under the CLSM. Hoechst 33258 (8 μg/mL) was used to stained the nuclei.

## RESULTS

### CyT selectively targeted RNA G4s by fluorescence light-up

**Figure 1.**
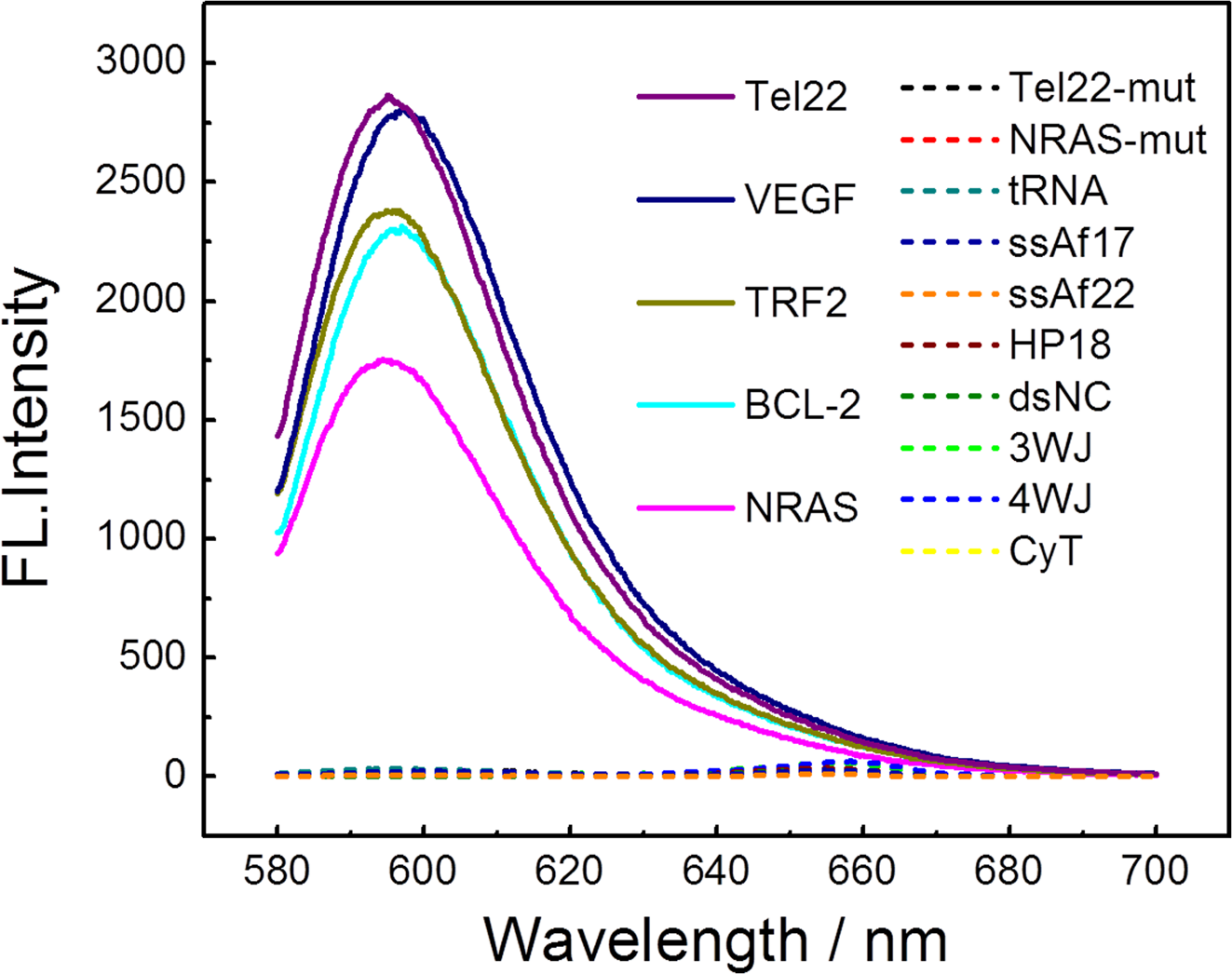
Fluorescence emission spectra of 2 μM CyT with various oligonucleotides (4 μM) in 20 mM Tris-HCl (40 mM KCl, pH 7.0) solution.

**Figure 2.**
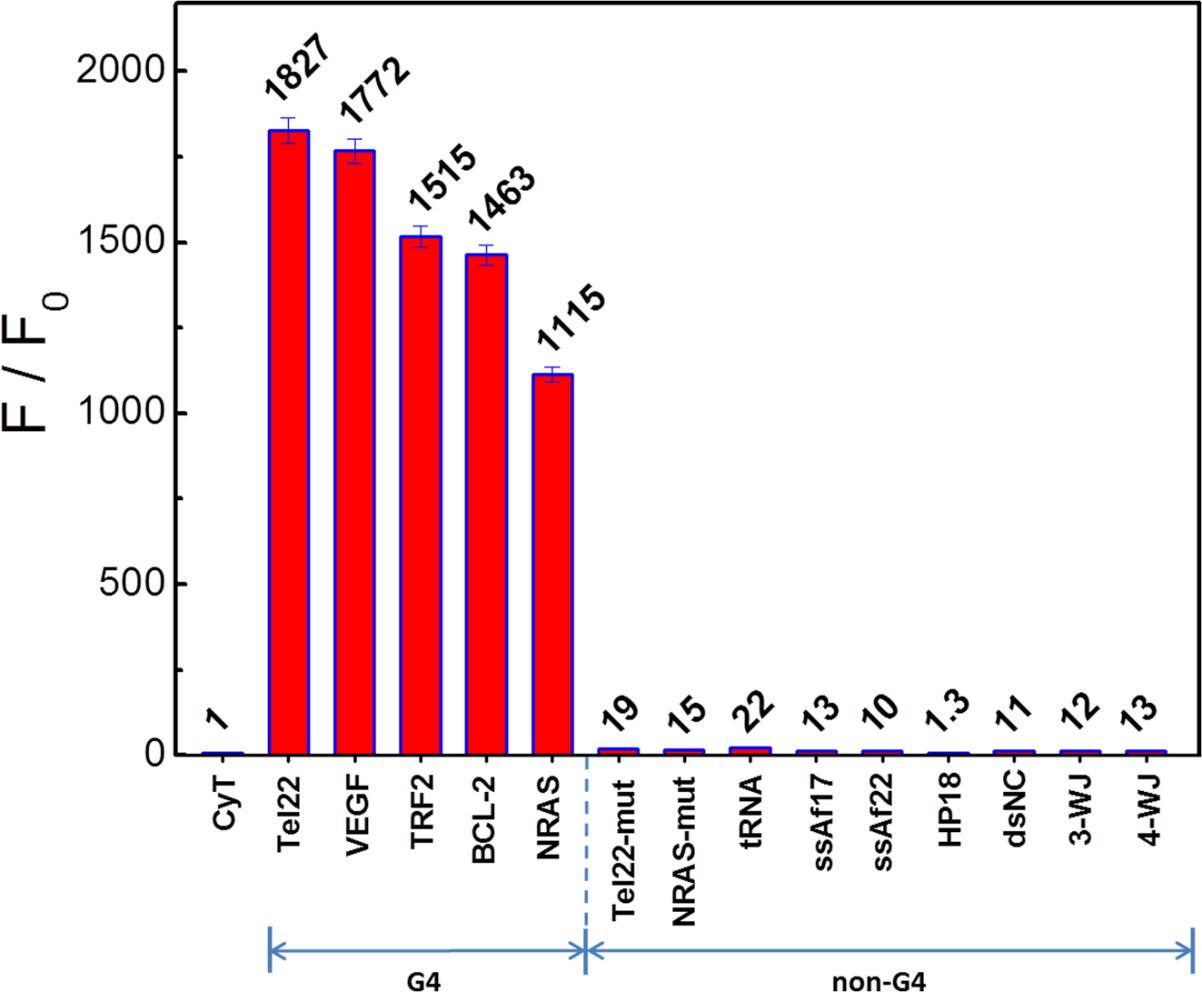
Dependence of CyT (2 μM) fluorescence intensity at 595 nm on a variety of RNA sequences (4 μM) in 20 mM Tris-HCl (40 mM KCl, pH 7.0) solution.

To demonstrate the feasibility of using a CyT probe for RNA G4 recognition, we compared the fluorescence intensity of CyT on its own or in the presence of various RNA sequences. The results of emission spectra are presented in Figure 1. Drastic fluorescence enhancements at 595 nm were observed by adding RNA G4s, such as Tel22, VEGF, TRF2, BCL-2 and NRAS. In contrast, CyT alone or in the presence of other RNA templates, such as single-stranded (ssAf17, ssAf22), hairpin (HP-18), double-stranded (dsNC), three-way junction (3-WJ), four-way junction (4-WJ), mutation sequences (Tel22-mut, NRAS-mut) and tRNA, induced very weak fluorescence upon interacting with CyT. As shown in Figure 2, surprisingly, in the presence of RNA G4 sequences (Tel22, VEGF, TRF2, BCL-2 and NRAS), the fluorescence enhancements at 595 nm were 1827-, 1772-, 1515-, 1463- and 1115-fold, respectively, which were markedly higher than with other RNA forms (1.3- to 22-fold). Based on these results, we conclude that CyT could distinguish RNA G4 structures from other RNA forms. Therefore, CyT is a strong candidate as a novel fluorophore that can selectively target general RNA G4 structures among other RNA forms.

Furthermore, fluorescence titrations were carried out to evaluate the binding constants of CyT with a series of RNA templates. A 1:1 binding model for CyT with RNA G4s (using VEGF as an example) was confirmed by the Job's plot analysis (Supplementary Figure S1). The typical titration curves and fitting results according to the 1:1 binding model are presented in Supplementary Figure S2. The binding constants for CyT with Tel22, VEGF, TRF2, BCL-2 and NRAS are (1.17 ± 0.37) × 10^5^, (1.44 ± 0.29) × 10^5^, (2.81 ± 0.63) × 10^5^, (1.94 ± 0.29) × 10^5^ and (3.49 ± 0.77) × 10^5^ M^−1^, respectively. In comparison with RNA G4s, the binding strength to tRNA, ssAf17, ssAf22, HP18 and dsNC was much weaker, with binding constants (6.35 ± 2.26) × 10^4^, (4.40 ± 0.38) × 10^4^, (4.03 ± 0.36) × 10^4^, (2.89 ± 0.28) × 10^4^ and (2.42 ± 0.25) × 10^4^ M^−1^, respectively. Of note, although the binding affinities of CyT to non-RNA G4s were obtained in the range of 10^4^ (Supplementary Table S1, CyT did not exhibit significant fluorescence enhancement. This may be due to the fact that CyT still has flexible orientations when binding to non-G4 structures. A previous study demonstrated that the binding affinity may not be a determining factor for enhanced fluorescence intensity (56).

As molar absorptivity and fluorescence intensity are key factors influencing the sensitivity of a fluorescence probe, we evaluated the molar extinction coefficients and quantum yields of CyT bound to RNA G4s. The CyT monomer showed a relatively high molar extinction coefficient of 80 14;000 M^−1^cm^−1^, while the fluorescence quantum yields of CyT bound to two equivalents of RNA G4s, such as Tel22, VEGF, TRF2, BCL-2 and NRAS, were 0.95, 0.92, 0.79, 0.74 and 0.58, respectively. The relatively high molar absorptivity and fluorescence intensity of the bound-state CyT may lead to CyT acting as a highly sensitive fluorescence probe for RNA G4 detection. Overall, the above results confirm that the CyT probe could be used for the recognition of RNA G4 structures with high specificity and sensitivity through fluorescence light-up.

### The structural state of RNA G4 significantly influenced the fluorescence response of CyT

**Figure 3.**
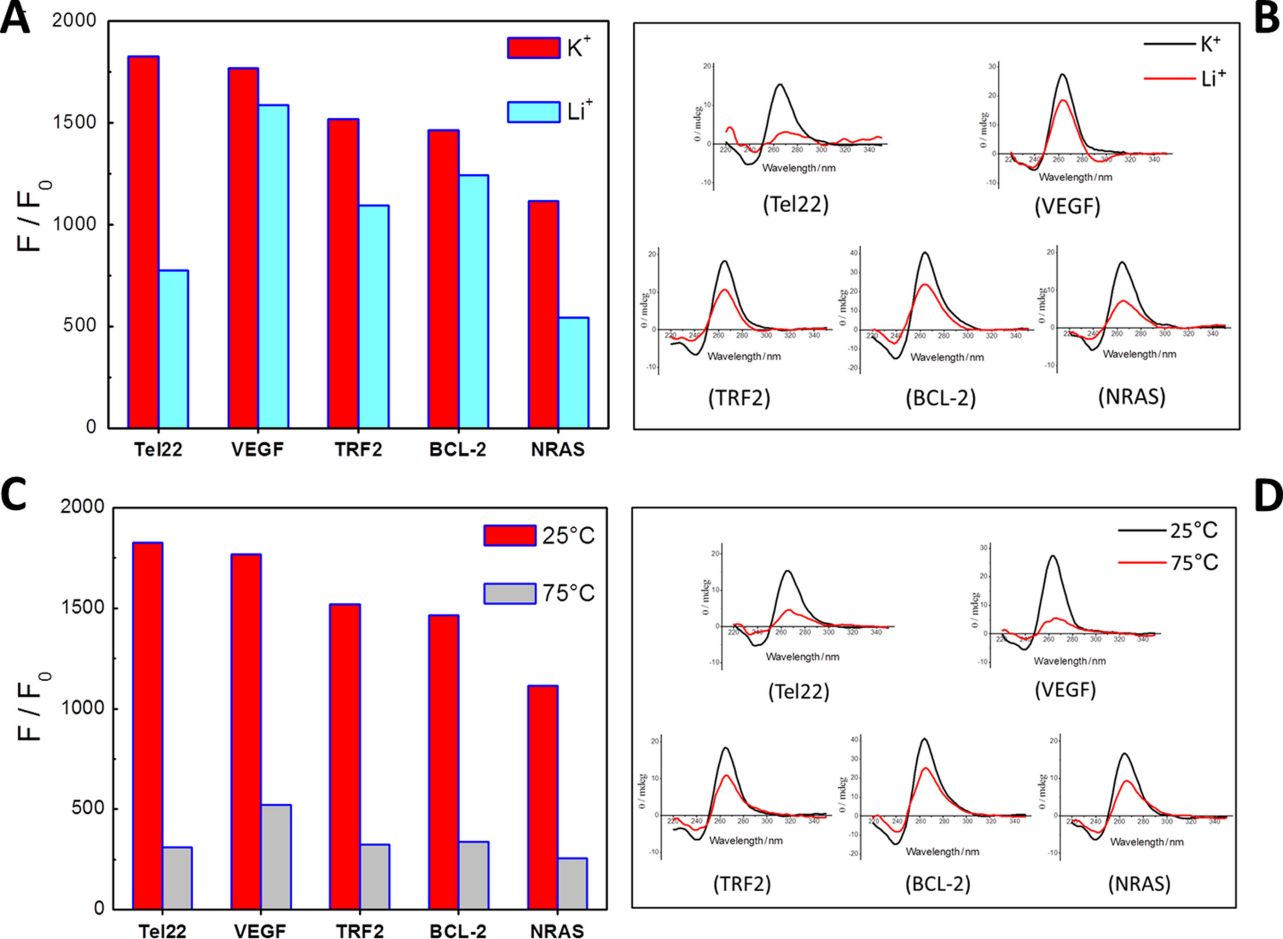
(**A**) Dependence of CyT (2 μM) fluorescence intensity at 595 nm on Tel22, VEGF, TRF2, BCL-2 and NRAS sequences (4 μM) in K^+^ and Li^+^ solutions. (**B**) Circular dichroism spectra of RNA G4 sequences (4 μM) in K^+^ and Li^+^ solutions. (**C**) Dependence of CyT (2 μM) fluorescence intensity at 595 nm on Tel22, VEGF, TRF2, BCL-2 and NRAS sequences (4 μM) at 25°C and 75°C. (**D**) Circular dichroism spectra of RNA G4 sequences (4 μM) at 25°C and 75°C.

As shown from the above results, CyT has a selective fluorescence response for RNA G4 structures, and therefore it was interesting to explore the relation between RNA G4 structure formation and the fluorescence response of CyT. Firstly, the fluorescence and CD spectra of VEGF, BCL-2, Tel22, TRF2 and NRAS sequences were measured in the presence of K^+^ and Li^+^ solutions. Figure 3A and B shows respectively the fluorescence intensity and CD spectra of G4 sequences under K^+^ and Li^+^ conditions. As shown in Figure 3B, a positive band at 264 nm and a negative band at 240 nm were observed in the presence of K^+^ and Li^+^, indicating the formation of parallel-type G4 structures. The CD intensity decreased under Li^+^ condition, compared to K^+^, which could be explained by the weaker G4 formation capability of Li^+^, compared to K^+^ (57,58). Furthermore, in presence of Li^+^, CyT emitted less fluorescence than with RNA G4 formed in presence of K^+^. These results show that the fluorescence intensity is also influenced by the cation present during G4 formation.

Additionally, binding assays of CyT in the presence of RNA G4 were also performed at different temperatures. The results (Figure 3C and D) show that, with the temperature increasing from 25 °C to 75 °C, a portion (and not whole) of RNA G4 structures was unfolded (as shown by the CD intensity), which lead to a decrease of CyT fluorescence.

Taken together, the above results show that CyT fluorescence is significantly influenced by the structural formation of RNA G4s, depending on the cations present and temperature. Therefore, this suggests that the CyT probe can sensitively and dynamically respond to the structural state of RNA G4s.

### Mechanism of recognition of RNA G4 structures by CyT

**Figure 4.**
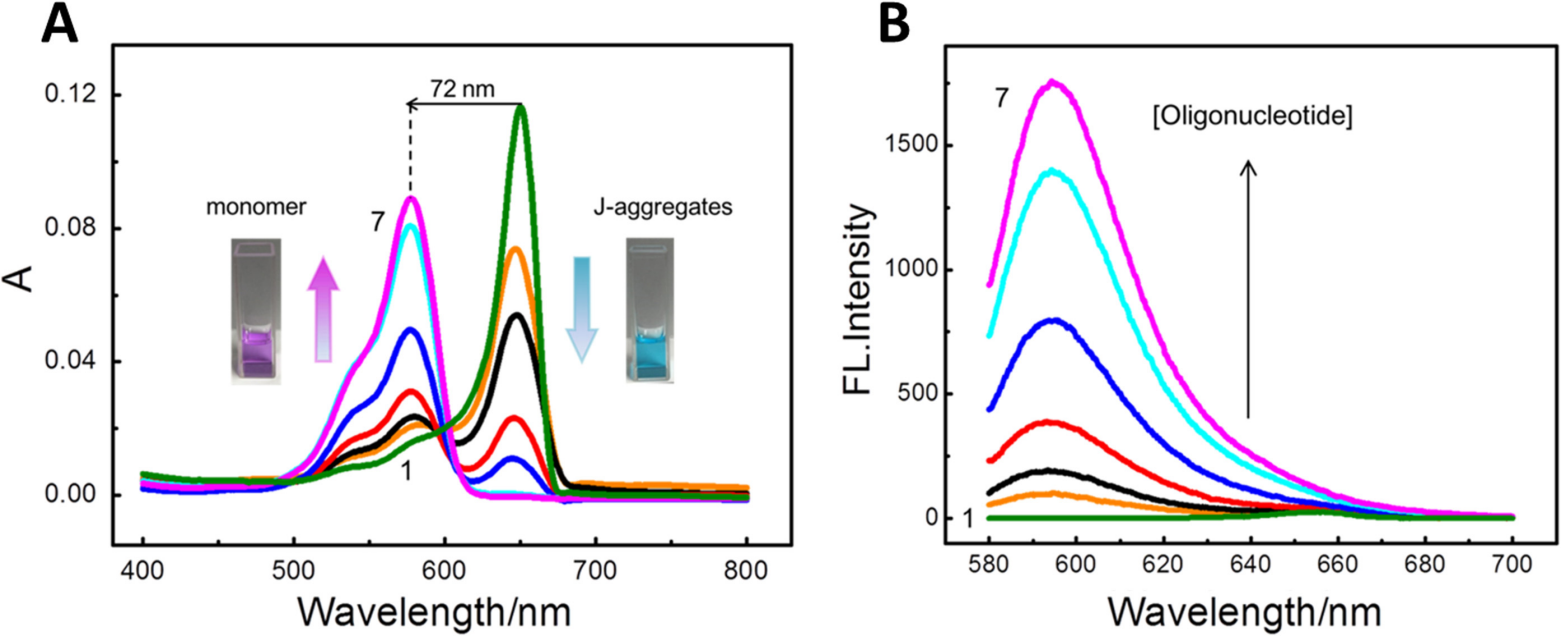
(**A**) Absorption spectra and (**B**) fluorescence emission spectra of CyT (2 μM) with RNA G4 sequence (NRAS) at seven concentrations (μM): (i) 0, (ii) 0.125, (iii) 0.25, (iv) 0.5, (v) 1, (vi) 2, (vii) 4.

To investigate the mechanism of recognition of RNA G4 structures by CyT, the interaction between CyT and RNA G4s (using NRAS as an example) was studied. A UV-vis titration was conducted by increasing the RNA G4 concentration (from 0.125 to 4 μM), with the CyT concentration fixed at 2 μM. As shown in Figure 4A, without any RNA G4 in the buffer solution, CyT self-assembled in Tris-HCl (K^+^) and displayed a predominant absorption band at 650 nm, which was previously assigned to J-aggregates (59). Of note, with increased NRAS concentrations, the J-aggregate band located at 650 nm gradually decreased and eventually disappeared when the ratio of [RNA]/[CyT] was 1, accompanied by the appearance of a new band located at 578 nm, which was previously ascribed to CyT J-aggregates disassembled to a monomer (59). These results showed that RNA G4 could disassemble CyT J-aggregates to a monomer, with a 72-nm blue-shift from that of J-aggregates. Next, the fluorescence intensity of CyT with different concentrations of NRAS was also examined (Figure 4B). The fluorescence intensity correlated with the concentration of RNA G4, which could be interpreted as the CyT monomer binding to RNA G4, thus hindering the rotation and inhibiting the radiationless transition. A significant enhancement of monomeric CyT fluorescence intensity was observed. Therefore, these results indicate that CyT recognizes RNA G4 by a dual-recognition mechanism, which comprises the disassembly of CyT J-aggregates and the binding of the CyT monomer to RNA G4 structures.

It is worth noting that the enhancement of monomeric CyT fluorescence intensity resulted from the bound-state CyT monomer, and not from the unbound-state monomer itself. As shown in Supplementary Figure S3, under methanol (unbound-state) and Tris-HCl (K^+^) (J-aggregates) conditions, the fluorescence intensity of CyT was very weak. In contrast, significant fluorescence enhancement was induced by the bound-state CyT monomer, which can be considered as a unique signature in the recognition of RNA G4 structures.

### CyT as an efficient probe for the detection of RNA G4s in a native gel system

**Figure 5.**
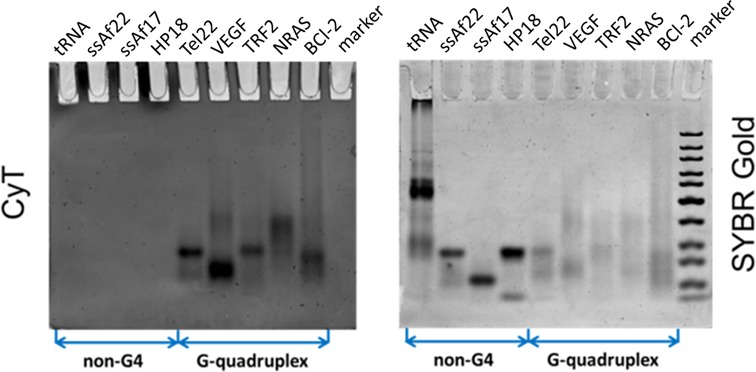
Recognition experiments on PAGE. RNA samples (5 μM) were prepared in 20 mM Tris-HCl buffer with 40 mM K^+^ and loaded on a non-denaturing 12% acrylamide gel. The gels were stained with (**A**) 20 μM CyT and (**B**) SYBR Gold, and visualized on GE Typhoon Trio.

To evaluate the ability of CyT to detect RNA G4s in various systems, a solution-based native PAGE experiment was performed whereby the RNA G4 structures were continuously maintained in the PAGE gel (60). As shown in Figure 5, CyT stained oligonucleotides Tel22, VEGF, TRF2, BCL-2 and NRAS, confirming that these sequences did indeed form RNA G4 structures that interacted with CyT. The four non-G4 RNA sequences, namely ssAf17, ssAf22, HP-18 and tRNA, did not interact with CyT. The staining intensities obtained from this experiment correlated well with the results of the fluorescence emission measurements shown in Figure 2. As a control, the positions of all RNAs were confirmed using SYBR Gold, a generic light-up probe for nucleic acids. Thus, the SYBR Gold probe detected the bands of all oligonucleotides. In contrast, CyT specifically recognized RNA G4s in the native gel system. Several oligonucleotides showed multiple bands, probably due to their multimeric and/or multiple structures (60).

**Figure 6.**
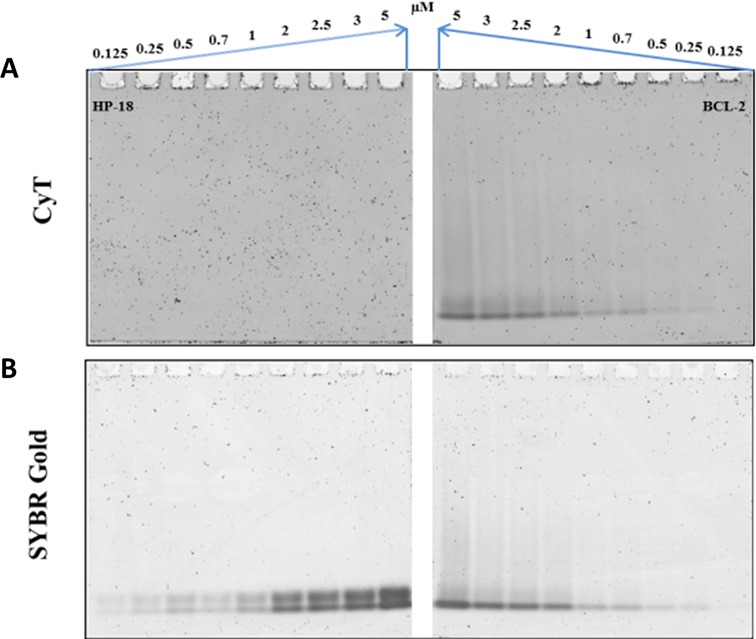
Experiment showing detection limits on native PAGE gels. Oligonucleotide sequences HP-18 (left panels) and BCL-2 (right panels) were tested at nine concentrations (0.125, 0.25, 0.5, 0.7, 1, 2, 2.5, 3 and 5 μM). RNA samples were prepared in 20 mM Tris-HCl buffer with 40 mM K^+^ and loaded on a non-denaturing 12% acrylamide gel. The 12% acrylamide gels (12%) were stained with either (**A**) 20 μM CyT or (**B**) SYBR Gold, and were visualized on GE Typhoon Trio.

To evaluate the effectiveness of CyT at various concentrations, the BCL-2 G4 sequence was retested in the gel system, along with the HP-18 sequence as negative control. In this experiment, oligonucleotides were loaded at nine different concentrations (0.125, 0.25, 0.5, 0.7, 1, 2, 2.5, 3 and 5 μM) and subsequently stained using either 20 μM CyT (Figure 6A) or SYBR Gold (Figure 6B). As shown in Figure 6A, no interactions with CyT, and thus no bands, were observed for the negative control sequence HP-18 at any concentration. On the other hand, a faint band was visible at 0.25 μM for the BCL-2 sequence, indicating an approximate detection limit of 3.75 ng. These results show CyT to be an excellent agent for detecting RNA G4 structures in gel systems, even at very low concentrations, with a level of sensitivity equal to that of other highly sensitive probes used for general RNA staining.

### CyT as a detection probe for non-canonical RNA G4 structures

Generally, the putative G4-forming sequence containing four tracts of three or more continuous guanines is prone to form a G4 structure by G-tetrads comprising a planar array of four guanine bases through hydrogen bonding interaction. However, recent research showed that nucleic acids that do not obey the classical description of sequences G_2+_N_L1_G_2+_N_L2_G_2+_N_L3_G_2+_ could also form G4 structures (21–24). Some sequences (such as Bulges-TB1 and Spinach), exhibiting discontinuous arrangement of guanines in the G-tetrad core, still form G4 structures (21,22), broadening the putative G4s in the genome. In addition, the tRNA-Cys and tRNA-Ala fragments (but not full-length tRNA) also assemble into G4 structures, further extending the application of G4 in biological functions. RNA G4 structures are associated with the regulation of transcription and translation. Thus, much research interest is focused on RNA G4 structures with a view to developing novel therapeutics, as RNA G4s have the potential to manipulate or influence biological processes. Therefore, an effective and universal G4 detector would be expected to have the ability to find more potential RNA G4-forming sequences that were undetected in earlier studies using bioinformatics searches. However, there have been few reports about molecular probes that can recognize and light up these non-canonical RNA G4 structures. Therefore, to investigate whether the CyT probe recognized non-canonical RNA G4 structures, we used the CyT probe to detect a series of non-canonical RNA G4s, including Bulges-TB1, tRNA-Cys fragment, tRNA-Ala fragment and Spinach, all of which were determined to be G4 structures by Mukundan and Phan (21), Ivanov *et al*. (54) and Warner *et al*. (22).

**Figure 7.**
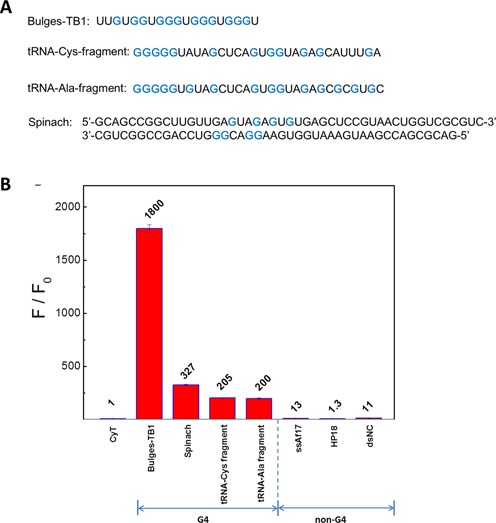
(**A**) Non-canonical G4 sequences. Guanine bases involved in the formation of RNA G4 structures are marked blue. (**B**) Dependence of CyT (2 μM) fluorescence intensity on noncanonical G4 sequences and nonG4 sequences (4 μM in 20 mM Tris-HCl, pH 7.0, 40 mM K^+^).

Four non-canonical RNA G4s, namely Bulges-TB1, Spinach, tRNA-Cys fragment and tRNA-Ala fragment (Figure 7A), were used to bind with the CyT probe, and single-stranded RNA (ssAf17), double-stranded RNA (dsNC) and hairpin RNA (HP-18) were used as nonG4 RNA controls. As shown in Figure 7B, the non-canonical RNA G4 sequences binding with CyT exhibited F/F_0_ fluorescence enhancement of about 1800-, 327-, 205- and 200-fold, respectively, which were higher than those obtained with ssAf17, dsNC and HP-18. Interestingly, the fluorescence enhancement was lower for some of the non-canonical RNAs than for the canonical ones. This may be due to non-canonical RNA G4 formation, with as many as three isolated guanines, exhibiting less stability, compared with canonical RNA G4s in which continuous G-tracts participated in the G-tetrad core formation. Although the light-up ability for non-canonical RNA G4s was not as strong as for canonical RNA G4s, the CyT was able to distinguish with precision non-canonical RNA G4s from other RNA forms. Based on these results, we propose that CyT could be developed as an effective and universal detector for more RNA G4 structures in the genome.

### DNA G4 probes (such as Thiazole Orange) may not be suitable for RNA G4 detection without validation

**Figure 8.**
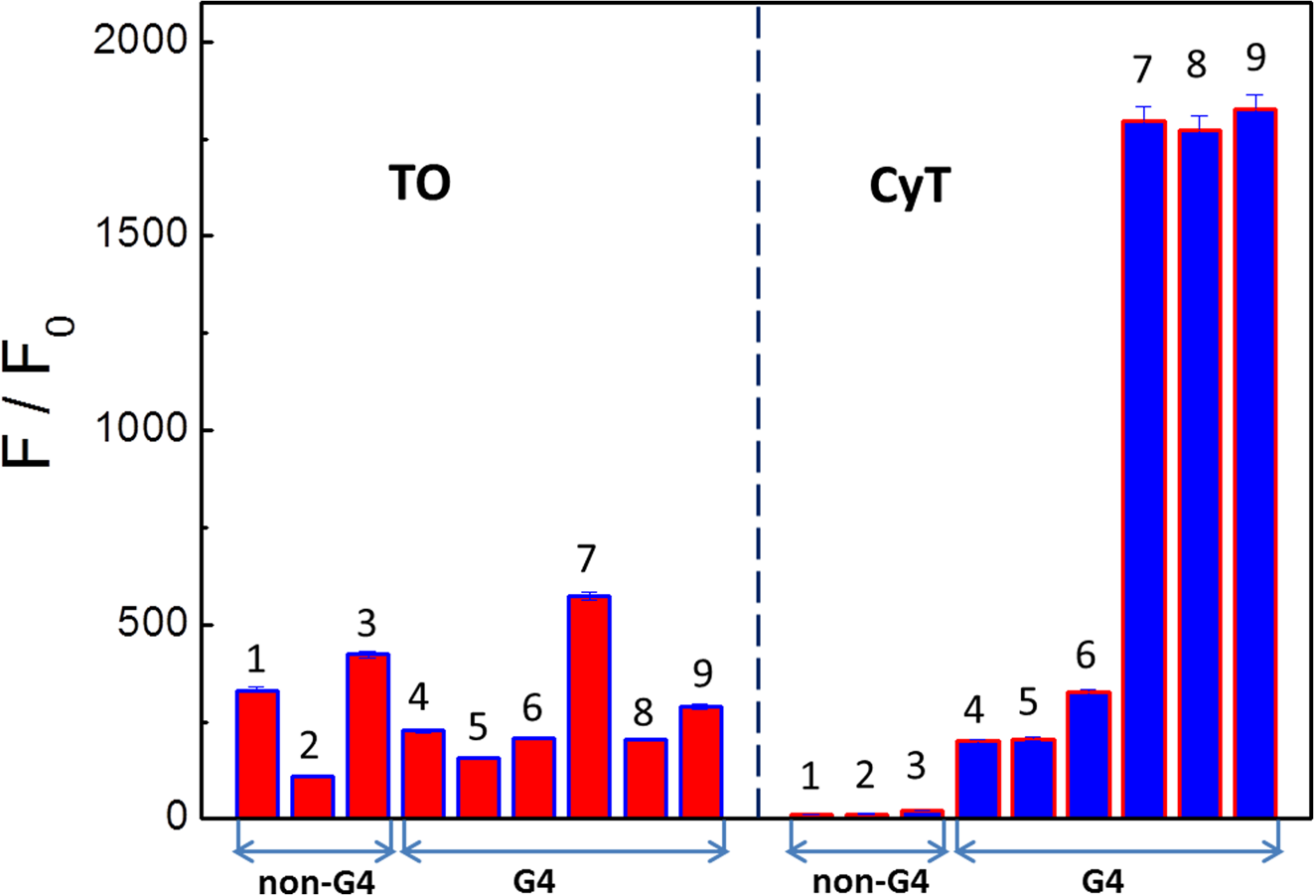
Dependence of TO and CyT fluorescence intensity at 595 nm on G4 and nonG4 sequences: (i) HP-18, (ii) ssAf17, (iii) tRNA, (iv) tRNA-Ala fragment, (iv) tRNA-Cys fragment, (vi) Spinach (vii) Bulges-TB1, (viii) VEGF and (ix) Tel22.

Given the structural similarities of DNA and RNA G4s, we investigated whether DNA G4 ligands also showed similar effects on RNA G4s by using TO, a well-known DNA G4-binding dye (31). Accordingly, we performed fluorescence assays for TO with different RNA forms. Canonical RNA G4 sequences (VEGF, Tel22), non-canonical RNA G4 sequences (RNA-Ala fragment, tRNA-Cys fragment, Spinach, Bulges-TB1), control sequences (HP-18, ssAf17) and tRNA were selected to interact with the TO probe. As shown in Figure 8, TO displayed fluorescence enhancement of about 330-, 100-, 420-, 230-, 150-, 200-, 570-, 200- and 290-fold toward (i) HP-18, (ii) ssAf17, (iii) tRNA, (iv) tRNA-Ala fragment, (v) tRNA-Cys fragment, (vi) Spinach, (vii) Bulges-TB1, (viii) VEGF and (ix) Tel22, respectively. These results show that, in contrast to CyT, the DNA G4 probe TO display no selectivity for RNA G4. Therefore, we conclude that a DNA G4 probe would not be suitable for detecting or recognizing RNA G4 without validation.

### Detecting RNA G4 in fixed human cells using CyT

**Figure 9.**
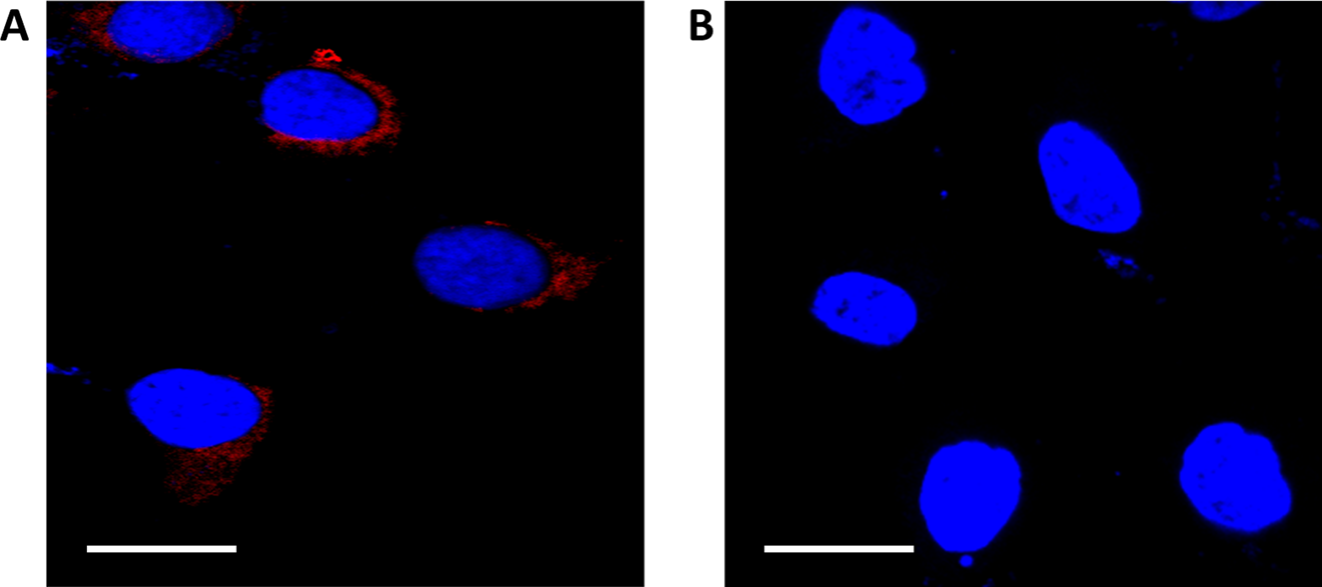
Confocal laser scanning microscopy showing binding of the fluorescence probe CyT with RNA G4s in the cytoplasm of A549 cells. Nuclei were colored blue by counterstaining with DAPI. Scale bars, 20 μM. (**A**) After treatment with the CyT probe (5 μM) for 5 min, red fluorescent foci were observed in the cytoplasm. (**B**) Loss of the red fluorescent foci in the cytoplasm after RNase T1 treatment.

Next, we explored the effectiveness of the CyT probe in detecting RNA G4s in human cells by using a CLSM method. Incubating fixed and permeabilized cells with the CyT probe resulted in red fluorescent foci in the cytoplasm, which were visible by CLSM (Figure 9A). In contrast, fixed and permeabilized cells treated with RNase T1 exhibited no foci in the cytoplasm (Figure 9B). Since RNase T1 splits RNA after guanosine residues, thus producing 3′-phosphorylated ends, the loss of CyT fluorescence in the cytoplasm is a consequence of the irreversible breakage of RNA G4 structures.

**Figure 10.**
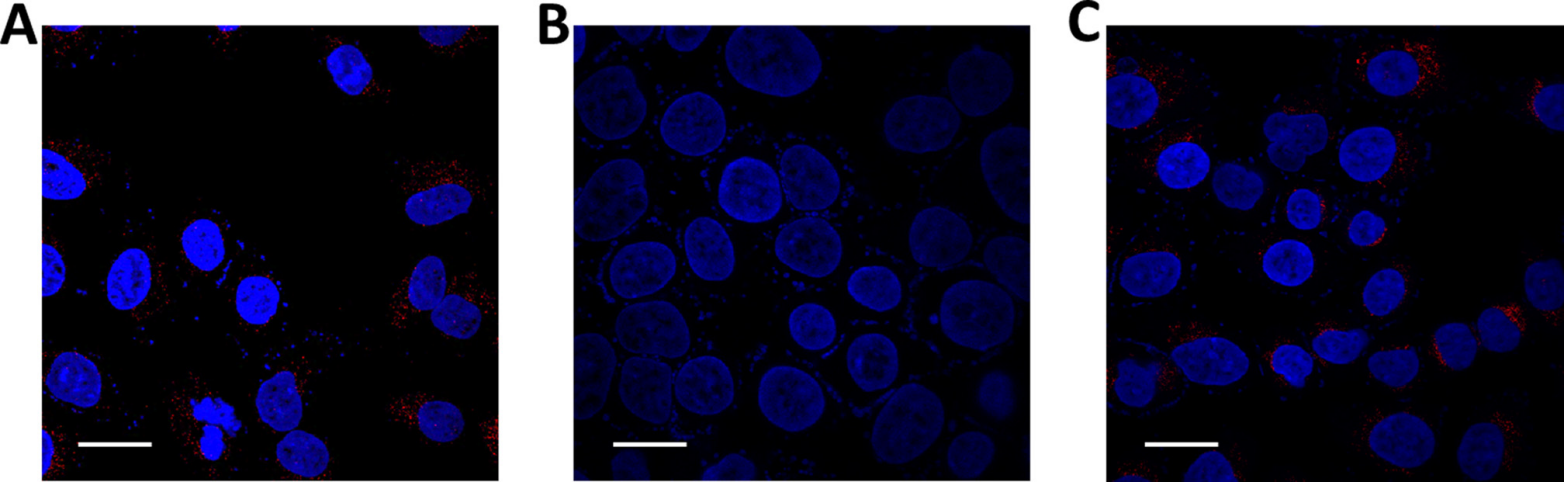
Confocal laser scanning microscopy showing binding of the CyT probe with RNA G4s in the cytoplasm of A549 cells. Nuclei were colored blue by counterstaining with DAPI. (**A**) After treatment with the CyT probe (5 μM) for 5 min, red fluorescent foci were observed in the cytoplasm.

Additionally, a urea-denaturing experiment was carried out to show that the CyT foci in the cytoplasm (Figure 10) were sensitive to the RNA G4 structure formation state. Since urea can destroy RNA secondary structures by forming stacking interactions and multiple hydrogen bonds with nucleic acid bases (61), the disappearance of the foci in the cytoplasm upon the addition of urea (Figure 10B) confirmed that the foci in the cytoplasm were indeed due to CyT binding to target RNA structures. Importantly, the RNA secondary structures were recovered when urea was flushed with PBS, as demonstrated by the reappearance of the fluorescence foci (Figure 10C). These results imply that CyT is sensitive to RNA structures and therefore could be used as an efficient probe to dynamically monitor the folding and unfolding of RNA G4s in the cytoplasm.

### Direct detection of RNA G4 in living human cells

It is well known that RNA G4 structures have a vital role in cell regulation processes and are associated with various forms of human cancers. A previous study demonstrated the existence of RNA G4 structures within the cytoplasm of human cells, using a G4 structure-specific antibody (4). Since the distribution of RNA G4s varies greatly not only within different cells, but also at the various stages of cancer, there is a great demand for an effective method to detect and locate endogenous RNA G4 structures in living cells.

**Figure 11.**
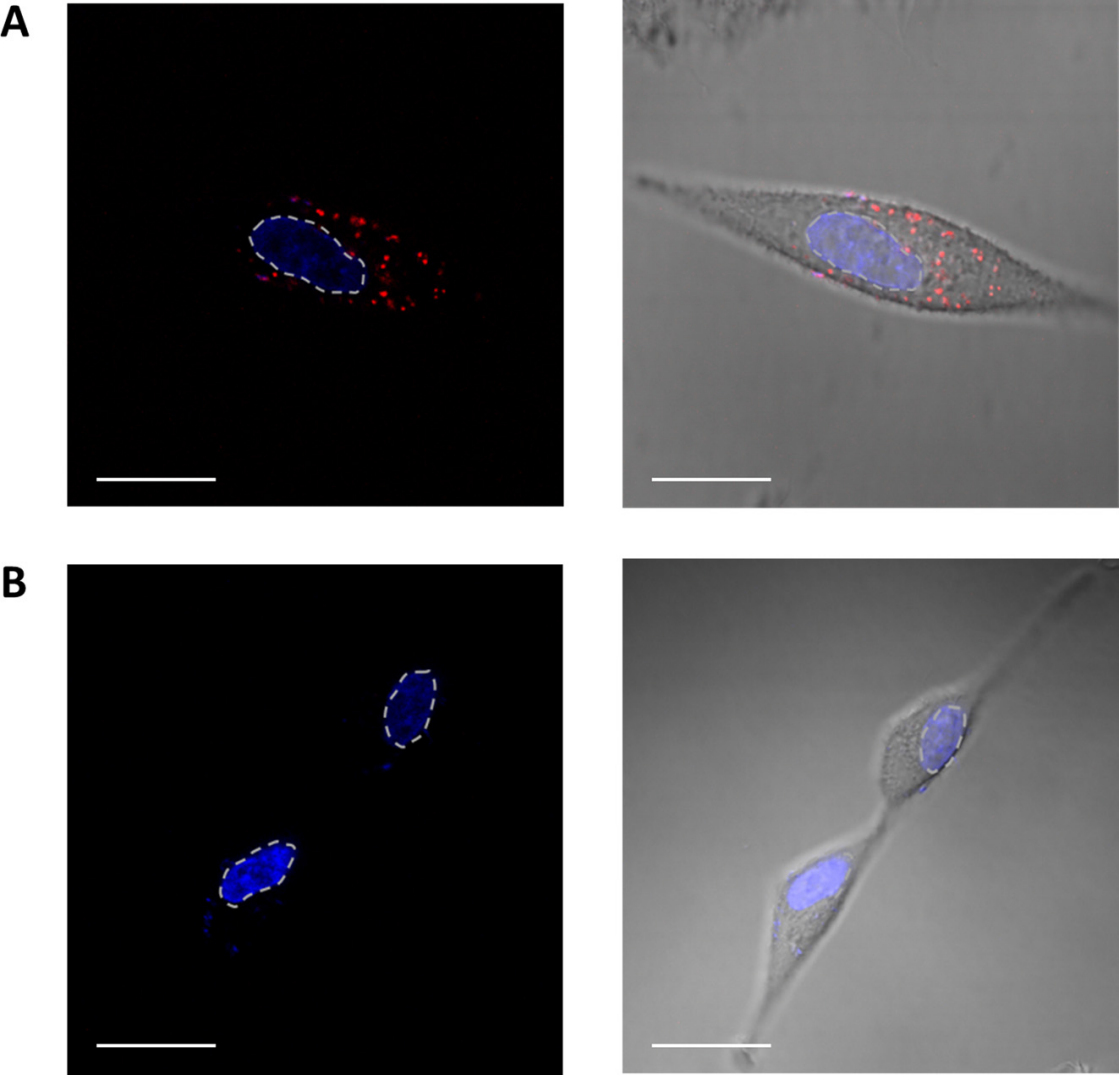
Confocal laser scanning microscopy showing binding of the fluorescence probe CyT with RNA G4s in the cytoplasm of A549 living cells. Nuclei were colored blue by counterstaining with the DNA dye Hoechst 33258, (**A**) with or (**B**) without incubation with the CyT probe (1.25 μM) for 24 h. The red channel shows CyT fluorescence in the cytoplasm, and the blue channel shows Hoechst 33258 for nuclear staining (the dotted lines indicate nuclear boundaries). Scale bar, 20 μM.

Although we demonstrated that the CyT probe resulted in cytoplasm foci on binding with RNA G4s (Figure 9A), it was necessary to provide a real-time RNA G4 detection method for living cells. To achieve this, human A549 cells were incubated with CyT (1.25 μM) for 24 h and then examined using CLSM. Results (Figure 11) show that the CyT probe effectively crosses the cell membrane to successfully bind to the RNA G4 structures in the cytoplasm. Localization of the endogenous RNA G4 structures was indicated by the fluorescence visible on the images. These results indicate that the light-up probe has the potential for real-time mapping of endogenous RNA G4 structures in a cellular environment, which would help further our understanding of the biological functions of these RNA structures.

## DISCUSSION

We consider that an ideal RNA G4 light-up probe should possess three characteristics: low self-fluorescence in the unbound state, strong fluorescence upon binding to RNA G4s and low fluorescence upon binding to non-G4 RNA forms. According to these standards, we discovered CyT, a cyanine dye with low self-fluorescence, which showed fluorescence enhancement of over 1000-fold in the presence of RNA G4s, compared to fluorescence enhancement of less than 25-fold observed in the presence of non-G4 RNA. The high selectivity of the CyT probe for RNA G4s is based on the low fluorescence background of J-aggregates and its low reactivity with any other RNA forms. Its dual-recognition mechanism allows for the establishment of an efficient method to detect novel RNA G4s and provides a promising tool for RNA G4-based biomarker discovery, with potential diagnostic applications. Interestingly, the CyT probe showed in the presence of RNA G4s much higher fluorescence than ETC, a similar probe that differs from CyT only by a methyl group, instead of an ethyl group (43),(Supplementary Figure S4). The logD evaluation of CyT and ETC revealed that ETC was more hydrophobic than CyT. As a result, ETC aggregates are more stable than CyT aggregates in hydrophilic solution (physiological buffer). Thus, the more stable ETC aggregates are not favored for G4 sensing. Additionally, as ETC and CyT stack to the G-tetrad in the G4 structure, the ethyl group in ETC disrupts the planarity of the molecule, and thus ETC is also not optimal for G4 binding.

In addition, the CyT assay we developed may be used to identify non-canonical RNA G4 formation. The common view on G4 formation is that continuous G-tracts are needed in the sequence, although recent research showed that nucleic acids obeying the classical description of sequences G_2+_N_L1_G_2+_N_L2_G_2+_N_L3_G_2+_ could also form G4 structures (21–24). However, there are only few reports about molecular probes that can recognize and light up these non-canonical RNA G4 structures. Therefore, an effective and universal detector would be expected to find new potential RNA G4-forming sequences that were undetected in earlier studies using bioinformatics searches. In this study, CyT was used to recognize non-canonical RNA G4 structures and showed fluorescence enhancement in some extent. The lower fluorescence obtained with non-canonical RNA G4 structures, compared to canonical RNA G4, may be due to non-canonical RNA G4, with as many as three isolated guanines, exhibiting less stability, compared to their canonical counterpart. Although with a weaker fluorescence enhancement, non-canonical RNA G4 structures are expected to be recognized by the CyT probe.

Generally, there are many forms of RNA structures in the cytoplasm, including single-stranded RNA (ssRNA), double-stranded RNA (dsRNA), short hairpin RNA (shRNA) and RNA junction structures. Fluorescence assays showed that CyT strongly lit up RNA G4 structures and barely lit up the tested single-stranded, double-stranded and hairpin RNA structures, tRNAs, and three- and four-way junction structures. As a result we conclude that CyT, with its high specificity for RNA G4, is an excellent probe to stain and light up RNA G4 structures, and not other RNA structures, in the cells.

Furthermore, given the structural similarities between DNA and RNA G4s, we determined whether DNA G4 detectors (such as TO) can also be applied as RNA G4 detectors. Although TO is widely used as a selective DNA G4 detector, our results showed that TO does not show any selectivity for RNA G4s, compared with other RNA forms. Therefore, this indicates that a DNA G4 probe may not be suitable for detecting or recognizing RNA G4s without validation.

Additionally, our results showed that CyT emitted stronger fluorescence in the presence of RNA G4s, compared with DNA G4s with identical sequences (Supplementary Figure S5). Although the CyT probe showed very high selectivity for RNA G4s, compared to other RNA forms, and thus can be used to distinguish RNA G4s from other RNA forms, CyT, at the present stage, may not be suitable for distinguishing RNA G4s and DNA G4s with different sequences.

The molecular probe CyT described in this study showed strong fluorescence enhancement in the presence of RNA G4 structures but did not show such a response toward other RNA forms. Therefore, our results demonstrate that CyT is an effective detector of RNA G4 structures both *in vitro* and in living human cells. The ability of CyT to distinguish between G4 and non-G4 RNA offers a promising tool for future RNA G4-based biomarker discovery, with potential diagnostic applications.

## Supplementary Material

SUPPLEMENTARY DATA
