# Supplementary material for: Directly lighting up RNA G-quadruplexes from test tubes to living human cells
Source: Nucleic Acids Res. 2015 Oct 17;43(20):9575–86. doi: 10.1093/nar/gkv1040 (PMC4787783; doi:10.1093/nar/gkv1040)
Supplement: SUPPLEMENTARY DATA [file supp_43_20_9575__index.html]

Directly lighting up RNA G-quadruplexes from test tubes to living human cells — Directly lighting up RNA G-quadruplexes from test tubes to living human cells — SUPPLEMENTARY DATA 

# Directly lighting up RNA G-quadruplexes from test tubes to living human cells

## SUPPLEMENTARY DATA

- SUPPLEMENTARY DATA
