# Supplementary material for: Directly lighting up RNA G-quadruplexes from test tubes to living human cells
Source: Nucleic Acids Res. 2015 Oct 17;43(20):9575–86. doi: 10.1093/nar/gkv1040 (PMC4787783; doi:10.1093/nar/gkv1040)
Supplement: SUPPLEMENTARY DATA [file supp_gkv1040_Supporting-Information-proof-final.docx]

Supplementary Data

**Directly lighting up the RNA G-quadruplexes from test tubes to living human cells**

Shujuan Xu^1,2^, Qian Li^1,*^, Junfeng Xiang^1^, Qianfan Yang^1^, Hongxia Sun^1^, Aijiao Guan^1^, Lixia Wang^1^, Yan Liu^1^, Lijia Yu^1,2^, Yunhua Shi^1,2^, Hongbo Chen^1^, and Yalin Tang^1,*^

^1^ National Laboratory for Molecular Sciences, Center for Molecular Sciences, State Key Laboratory for Structural Chemistry of Unstable and Stable Species, Institute of Chemistry Chinese Academy of Sciences, Beijing, 100190, P. R. China

^2^ University of the Chinese Academy of Sciences, Beijing, 100049, P. R. China

* To whom correspondence should be addressed. Tel: + 86 10 62522090; Fax: +86 10 62522090; Email: tangyl@iccas.ac.cn. Correspondence may also be addressed to Qian Li, Tel: + 86 10 62558322; Fax: +86 10 62558322; Email: qianlee@iccas.ac.cn.

**Contents**

| **Part 1 Identification of CyT** | **S2-S3** |
| --- | --- |
| **Part 2 Determination of binding constants by fluorescence titration experiments** | **S4-S5** |
| **Part 3 Fluorescence spectra of CyT in different states** | **S6** |
| **Part 4 Fluorescence intensity enhancement of CyT and ETC with RNA G4s** | **S7** |
| **Part 5 Fluorescence intensity enhancement of CyT with RNA and DNA G4s** | **S8** |

**Part 1 Identification of CyT**

**a) Mass Spectrum**

*MS(ESI-Q-TOF) m/z [M-H]^-^ = 665.45

The calculated exact mass of C_32_H_29_N_2_O_6_S_4_^-^ is 665.09.

**b) Elemental Analysis**

Molecular formula of CyT: C_38_H_45_N_3_O_6_S_4_·3H_2_O

| Element | N | C | H | S |
| --- | --- | --- | --- | --- |
| Calculated Value (%) | 5.11 | 55.52 | 6.25 | 15.60 |
| Experimental Value (%) | 5.00 | 55.33 | 6.20 | 15.96 |

**c) NMR spectrum**

The numbering scheme of cyanine dye CyT.





The ^1^H-NMR spectrum of CyT in DMSO-d6


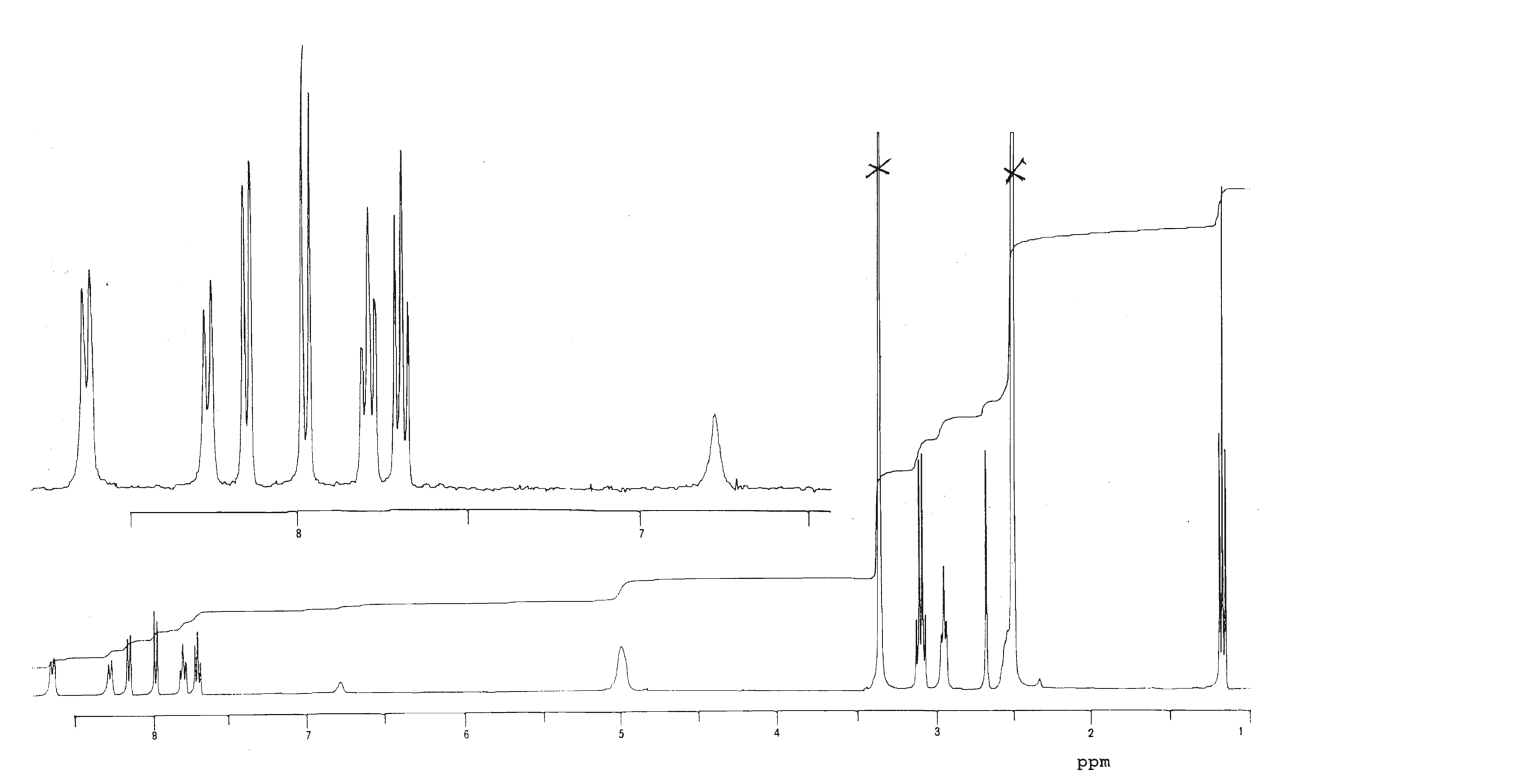
The full assignments of the proton peaks of CyT

| Proton number | ^1^H peak | Proton number | ^1^H peak |
| --- | --- | --- | --- |
| 1 | 8.16-8.14, d | 13 | 7.70, t |
| 2 | 7.80, t | 14 | 7.80, t |
| 3 | 7.70, t | 15 | 8.16-8.14, d |
| 4 | 7.99-7.97, d | 16 | 2.96, t |
| 5 | 8.28-8.26, d | 17 | --* |
| 6 | 8.64-8.62, d | 18 | 4.99, s |
| 7 | 6.777, s | 19 | 2.96, t |
| 8 | 2.685, s | 20 | --* |
| 9 | 6.777, s | 21 | 4.99, s |
| 10 | 8.64-8.62, d | 22 | 3.13-3.07, q |
| 11 | 8.28-8.26, d | 23 | 1.17, t |
| 12 | 7.99-7.97, d |  |  |

* The peaks of these protons are covered by the peak of the solvent DMSO-d6

**Part 2 Determination of binding constants by fluorescence titration experiments**

**
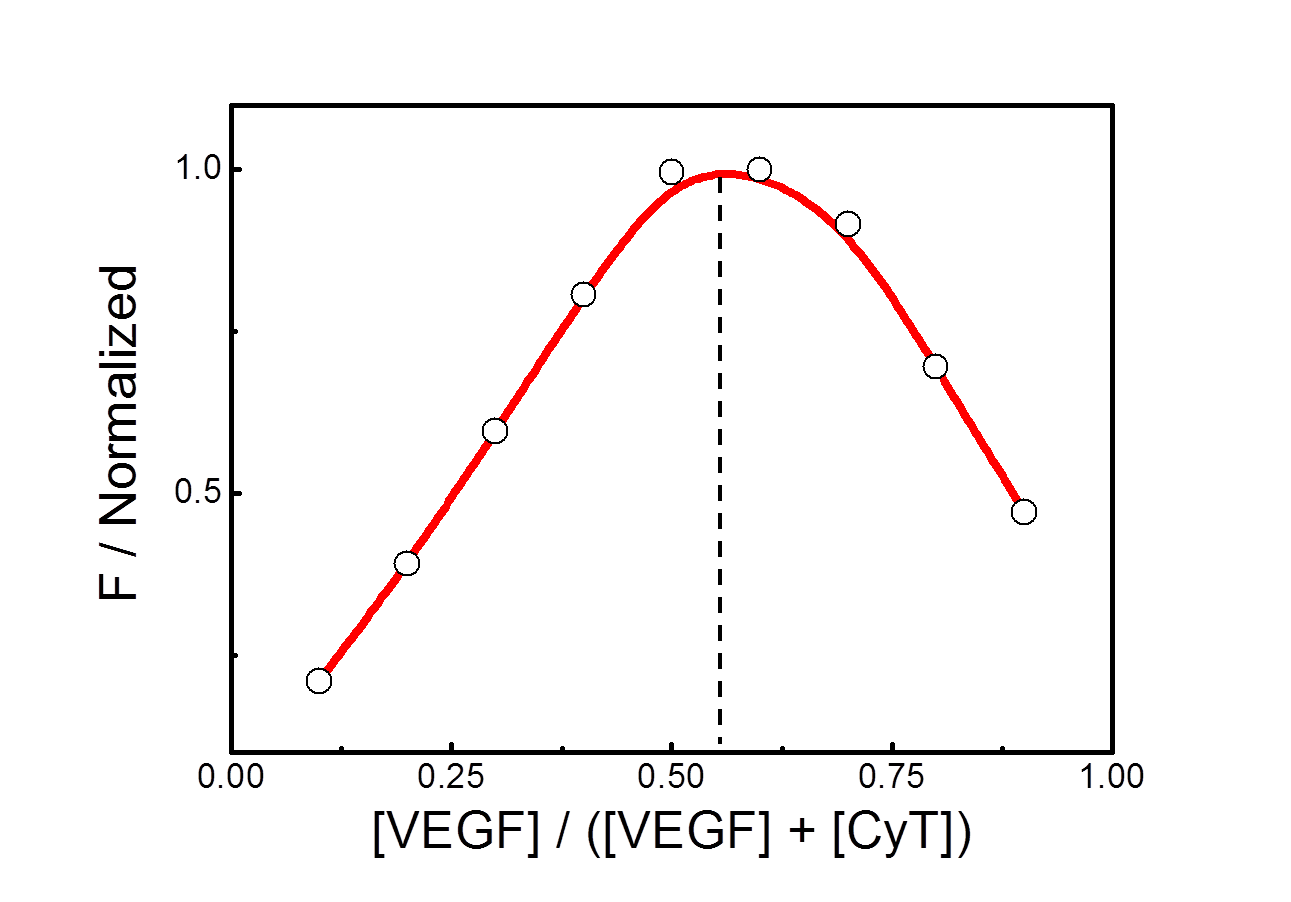
**

**Figure S1.** Job’s plot analysis of the stoichiometry of VEGF binding to CyT.


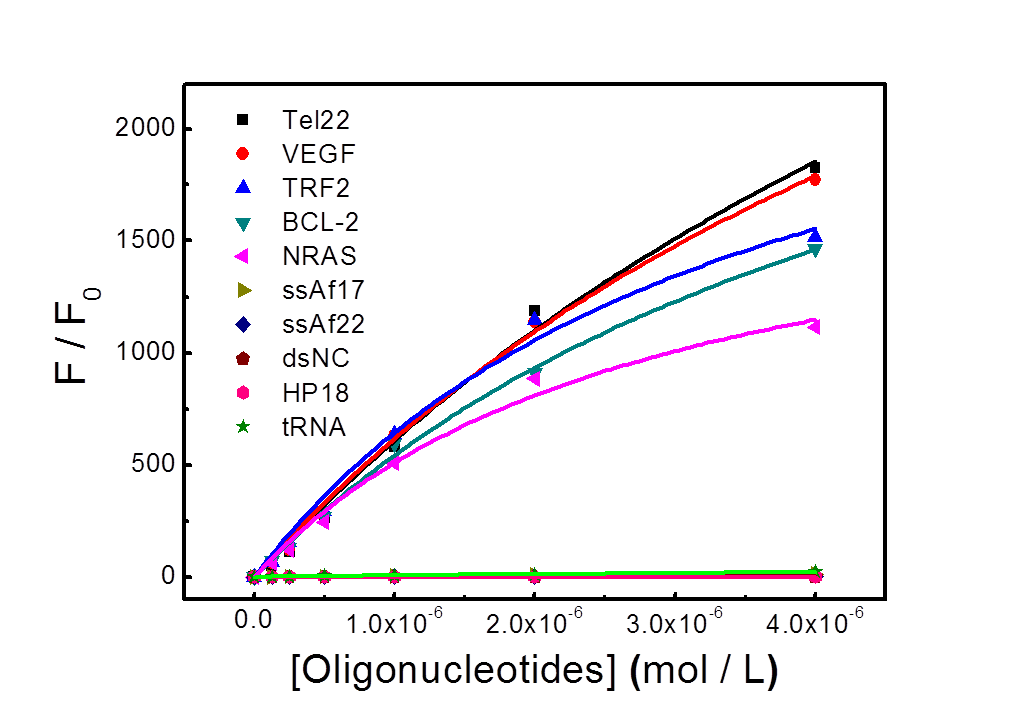


**Figure S2.** Binding curves at 595 nm for CyT (2µM) with different RNAs in 20 mM Tris-HCl buffer (pH 7.0) containing 40 mM K^+^. The solid lines are the fitted curves assuming 1:1 stoichiometry.

**Table S1.** Binding constants for the CyT probe with different RNA forms determined from the fitted curves.

| RNA sequences | Binding constants |
| --- | --- |
| Tel22 | (1.17 ± 0.37) × 10^5^ M^-1^ |
| VEGF | (1.44 ± 0.29) × 10^5^ M^-1^ |
| TRF2 | (2.81 ± 0.63) × 10^5^ M^-1^ |
| BCL-2 | (1.94 ± 0.29) × 10^5^ M^-1^ |
| NRAS | (3.49 ± 0.77) × 10^5^ M^-1^ |
| tRNA | (6.35 ± 2.26) × 10^4^ M^-1^ |
| ssAf17 | (4.40 ± 0.38) × 10^4^ M^-1^ |
| ssAf22 | (4.03 ± 0.36) × 10^4^ M^-1^ |
| HP18 | (2.89 ± 0.28) × 10^4^ M^-1^ |
| dsNC | (2.42 ± 0.25) × 10^4^ M^-1^ |

**Part 3** **Fluorescence spectra of CyT in different states**


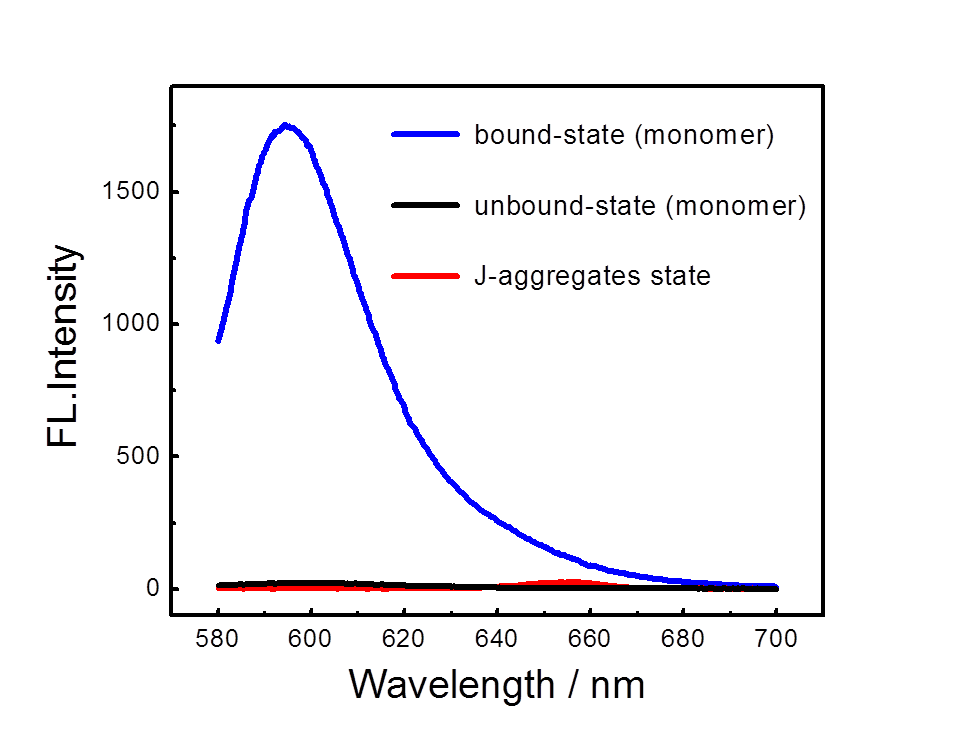


**Figure S3.** Fluorescence spectra of CyT in different states

**Part 4 Emission intensity enhancement of CyT and ETC with RNA G4s**


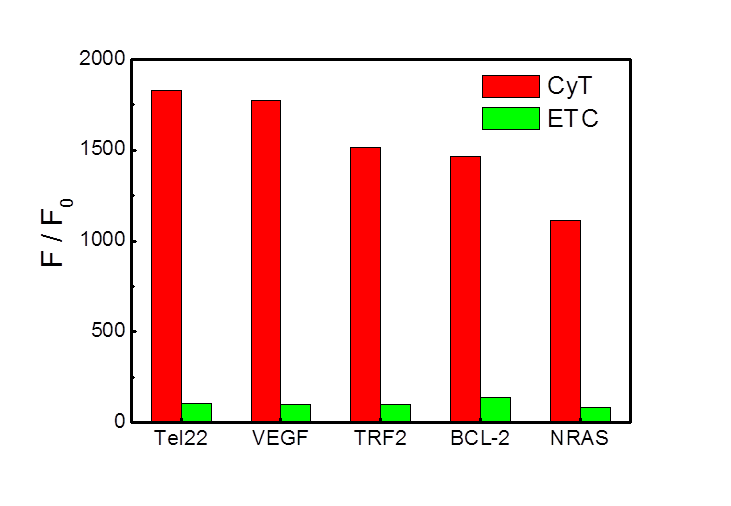


**Figure S4.** Comparison of emission intensity enhancement in CyT and ETC. Emission intensity of CyT and ETC (2 μM) mixed with 4 μM RNAs. All Samples were prepared at 20 mM Tris-HCl buffer (pH 7.0) containing 40 mM K^+^.

**Part 5** **Emission intensity enhancement of CyT with RNA and DNA G4s**
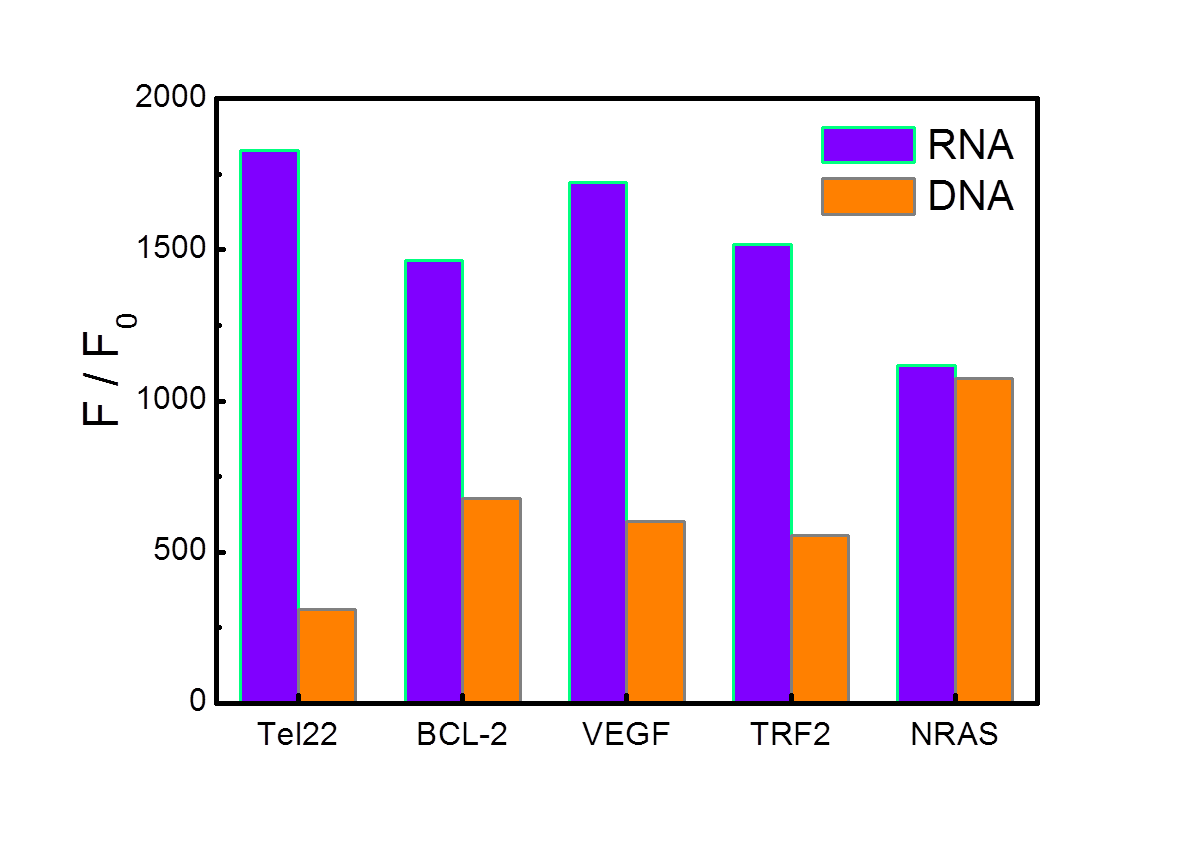


**Figure S5**. Fluorescence intensity at 595 nm on the RNA G4 sequences and DNA counterparts of the RNA G4 in 20 mM Tris-HCl (40 mM K^+^, pH=7.0) solution.

DNA counterparts used in this work: Tel22: AGGGTTAGGGTTAGGGTTAGGG, BCL-2: AGGGGGCCGTGGGGTGGGAGCTGGGG, VEGF: GGAGGAGGGGGAGGAGGA, TRF2: CGGGAGGGCGGGGAGGGC, NRAS: GGGAGGGGCGGGTCTGGG.
